# Annotations

**Published:** 1888-02-11

**Authors:** 


### ANNOTATIONS.

A Medical Philosopher.

DR. HUTCHISON STIRLING, F.R.C.S.E., has been appointed *Gifford* Lecturer on Natural Theology, and will probably enter upon his duties in the course of the year. It is not often that medical men devote themselves to metaphysical philosophy, which is perhaps rather surprising, considering the nature of their studies in connexion with the human and comparative anatomy and physiology of the brain. The choice of Dr. Stirling as the first holder of the valuable and unique *Gifford Lectureship* is one which will be decidedly gratifying to the more thoughtful and liberal-minded members of the medical profession. It cannot be said that the eminent physicians and surgeons of recent years have done much to establish the reputation of medicine for academic culture. In everything that relates to mere medicine and surgery, modern English practitioners have displayed a zeal and capacity worthy of the best traditions of their art. But in the wider regions of speculative philosophy and the more universally interesting fields of general literature modern medical men are hardly known. The want of a broader literary culture gives a certain narrowness and a somewhat illiberal professionalism, to many even of the most eminent physicians and surgeons of the present time, and sometimes renders their judgment, on ethical and other questions outside the range of strictly technical subjects, curiously unlike that of men accustomed to a profounder and wider range of thought. This is rather mortifying to the younger and more cultured men, and they are sometimes made to feel that, compared with eminent ecclesiastics and lawyers, doctors are conspicuously out of the running in the matter of liberal culture. The appointment of Dr. Hutchison Stirling is a redeeming circumstance, and one calculated both to console and inspire those who are jealous for the honour of their own profession of medicine. Dr. Stirling will enjoy a very substantial emolument, approaching, we believe, two thousand per annum. He has for some time given up the practice of medicine, which he followed many years ago as a general practitioner in Wales. He is well known in philosophical circles as the author of "The Secret of Hegel," and as a profound student of Kant.

A Scientific Preacher.

PREACHING on Sunday week on behalf of the Keighley Cottage Hospital, the Rev. E. Pringle, whose sermon is reported at length in the *Keighley News*, said: "There is a good deal of pious and very foolish talk about sickness and affliction generally. People speak as if God sent disease and sickness. It only requires a little thought on our part to find out how mistaken such a notion is. If a man slips on the ice and breaks his leg, you can hardly say God has afflicted him. Or if a man goes out on a raw, foggy night badly defended against the cold, you cannot say God has given that man pleurisy or bronchitis. Is God any more concerned in the origin and spread of fever or small-pox or of diseases that are inherited. . . . Speaking of bodily disorder as a consequence of disregarded law, it is needful to remember that here, as in other departments of life, the innocent often suffer. Many of the constitutional and hereditary disorders from which one generation suffer might be traced to the excesses or neglects of former generations. The strange susceptibilities and predispositions to certain ailments, many so-called nervous complaints and unaccountable complications of disorders that afflict one's fellow-creatures to-day have really a long history—a history in some cases beginning many lives back. The consequences of neglect, or of ignorance of the plain conditions of well-being, have been accumulating until they finally make their discharge on this or that poor mute and wondering victim. . . . When we think of the hardships and privations—the bare homestead, the depressed energies, the lost hopefulness, the unwelcome but unavoidable debt, and the endangered self-respect which so often follow the illness of a poor man, it seems there is a matter over and above the maintenance of hospitals and convalescent homes—and that is the prevention of sickness. There is a duty in regard to prevention which is not recognised as it ought to be. In some quarters a right opinion is yet to be created." We have quoted at some length because we are decidedly of opinion that among the masses of the people the merest elements of sanitary science are unknown or despised. Clergymen, above all people, have unique opportunities for the dissemination of sound general principles on these profoundly important subjects. It would be of great service to the community if they would from time to time depart from the beaten track of pulpit exhortations, and instruct their hearers on the much-neglected "religion of the body."

The Intellectual Woman of the Period.

THE falling off in the yearly number of marriages in these islands is causing anxiety in many bosoms, from those of the social scientists to those of fashionable dressmakers. Male contributors to the discussion raised by recent statistics have, in the fulness of their kindly hearts, drawn piteous pictures of Phyllis sitting sad and lonely in her bower because Corydon will not give up his pipe, his club, and other manly joys to make her his bride. Poor souls! they meant no harm, but Phyllis is indignant with them, and a lady, writing to a provincial contemporary, depicts the modern intellectual woman as more than contented with the sweet companionship of Euclid and Darwin (in cloth 8vo.), and scornfully indifferent to "the profession of a matron." "It is certainly not true," she says, "that all women are waiting with 'bated breath and whispering humbleness' for an advantageous offer of marriage." Again, "The great majority of mothers can be mothers only. Of such women, it is no exaggeration to say that their existence is only a disguised slavery. If marriage, then, is becoming unpopular, it is not merely from indisposition on the part of men, but from growing aversion to it on the part of women. Marriage for educated women is now only one of many possible occupations, and educated women may be excused if they regard it as the least desirable of them." So says our Newcastle lady, and we do not for a moment doubt the accuracy of her statements. There is a large and increasing number of women who despise marriage and motherhood, and look upon the duties these involve as merely burdensome, if not degrading. This is so; and it is a thing deeply to be regretted. For the ideal these women have set up is, in the first place, a selfish ideal. They are more concerned to gain another fragment of intellectual information (which they mis-name culture), and to do work which will bring them the applause of the world, than to fulfil those natural duties which bring into play woman's natural patience and love of detail. They live for themselves only—self-centred, self-opiniated, self-satisfied; and so dense are they that they fail to perceive as—not only simpler, but more intellectual—women do, the noble side of functions which they look at in their merely physical aspect. It is also a false ideal. Culture does not come from books alone, these must be supplemented by the lessons of life, which no woman fully learns except as wife and mother. We are desirous that all our women should be well-educated, intellectual, cultured, not because, as the Newcastle lady says, they won't, but because we hope they will marry. A wife and mother cannot have too much culture; and indeed, it will generally be found that our most distinguished intellectual and progressive women regard the positions which nature calls on women to occupy with the deepest reverence. It is sad to think how many of our girls, capable of higher and better things, are straying in the arid paths of a false intellectualism, mistaking "blue"-ness for culture, introspection for superior sensitiveness, and the mirage of posing before the world for the solid facts of a citizen's duty. We would not have them study less, but live more largely. Meanwhile, we commend to them the study of "Aurora Leigh," or still better, some practical experience that "art is much, but love is more."

